# Association of a Geriatric Emergency Department Innovation Program With Cost Outcomes Among Medicare Beneficiaries

**DOI:** 10.1001/jamanetworkopen.2020.37334

**Published:** 2021-03-01

**Authors:** Ula Hwang, Scott M. Dresden, Carmen Vargas-Torres, Raymond Kang, Melissa M. Garrido, George Loo, Jeremy Sze, Daniel Cruz, Lynne D. Richardson, James Adams, Amer Aldeen, Kevin M. Baumlin, D. Mark Courtney, Stephanie Gravenor, Corita R. Grudzen, Gloria Nimo, Carolyn W. Zhu

**Affiliations:** 1 Department of Emergency Medicine, Yale University, New Haven, Connecticut; 2 Geriatric Research, Education Clinical Center, James J. Peters Veterans Affairs Medical Center, Bronx, New York; 3 Department of Emergency Medicine, Northwestern University Feinberg School of Medicine, Chicago, Illinois; 4 Department of Emergency Medicine, Icahn School of Medicine at Mount Sinai, New York, New York; 5 Center for Healthcare Studies, Northwestern University, Chicago, Illinois; 6 Department of Health Law, Policy and Management, Boston University School of Public Health, Boston, Massachusetts; 7 Partnered Evidence-Based Policy Resource Center, Boston VA Healthcare Systems, Boston, Massachusetts; 8 Department of Emergency Medicine, New York University Grossman School of Medicine, New York; 9 Department of Population Health, New York University Grossman School of Medicine, New York; 10 US Acute Care Solutions, Canton, Ohio; 11 Department of Emergency Medicine, Perelman School of Medicine at the University of Pennsylvania, Philadelphia; 12 Department of Emergency Medicine, The University of Texas Southwestern Medical Center, Dallas; 13 Medecipher, Denver, Colorado

## Abstract

**Importance:**

There has been a significant increase in the implementation and dissemination of geriatric emergency department (GED) programs. Understanding the costs associated with patient care would yield insight into the direct financial value for patients, hospitals, health systems, and payers.

**Objective:**

To evaluate the association of GED programs with Medicare costs per beneficiary.

**Design, Setting, and Participants:**

This cross-sectional study included data on Medicare fee-for-service beneficiaries at 2 hospitals implementing Geriatric Emergency Department Innovations in Care Through Workforce, Informatics, and Structural Enhancement (GEDI WISE) (Mount Sinai Medical Center [MSMC] and Northwestern Memorial Hospital [NMH]) from January 1, 2013, to November 30, 2016. Analyses were conducted and refined from August 28, 2018, to November 20, 2020, using entropy balance to account for observed differences between the treatment and comparison groups.

**Interventions:**

Treatment included consultation with a transitional care nurse (TCN) or a social worker (SW) trained for the GEDI WISE program at a beneficiary’s first ED visit (index ED visit). The comparison group included beneficiaries who were never seen by either a TCN or an SW during the study period.

**Main Outcomes and Measures:**

The main outcome evaluated was prorated total Medicare payer expenditures per beneficiary over 30 and 60 days after the index ED visit encounter.

**Results:**

Of the total 24 839 unique Medicare beneficiaries, 4041 were seen across the 2 EDs; 1947 (17.4%) at MSMC and 2094 (15.4%) at the NMH received treatment from either a GED TCN and/or a GED SW. The mean (SD) age of beneficiaries at MSMC was 78.8 (8.5) years and at NMH was 76.4 (7.7) years. Most patients at both hospitals were female (6821 [60.8%] at MSMC and 8023 [58.9%] at NMH) and White (7729 [68.9%] at MSMC and 9984 [73.3%] at NMH). Treatment was associated with statistically significant mean savings per beneficiary of $2436 (95% CI, $1760-$3111; *P* < .001) at one ED and $2905 (95% CI, $2378-$3431; *P* < .001) at the other ED in the 30 days after the index ED visit. The association between treatment and mean cumulative savings at 60 days after the index ED visit per beneficiary was also significant: $1200 (95% CI, $231-$2169; *P* = .02) at one ED and $3202 (95% CI, $2452-$3951; *P* < .001) at the other ED.

**Conclusions and Relevance:**

Among Medicare fee-for-service beneficiaries, receipt of ED-based geriatric treatment by a TCN and/or an SW was associated with lower Medicare expenditures. These estimated cost savings may be used when calculating or considering the bundled value and potential reimbursement per patient for GED care programs.

## Key Points

**Question:**

Is there an association between geriatric emergency department (ED) programs and total costs of care for Medicare?

**Findings:**

In this cross-sectional study of 24 839 Medicare fee-for-service beneficiaries at 2 EDs, there was a significant association with reduced total costs of care after being seen by either a transitional care nurse and/or social worker trained to deliver geriatric emergency care. Per beneficiary, these savings were as much as $2905 after 30 days and $3202 after 60 days of the index ED visit.

**Meaning:**

These findings suggest that geriatric emergency department care programs may be associated with savings value to hospitals and payers.

## Teaser

This cross-sectional study assesses the association of geriatric emergency department program implementation with Medicare costs per beneficiary.

## Introduction

With the aging of the US population and increase in older adults requiring medical care across multiple settings, there has been greater interest in the dissemination and implementation of geriatric emergency care programs. The conceptual model of geriatric emergency departments (GEDs) was first proposed in 2007,^1^ when no such designation existed. The first GED accreditation program was created in 2018 by the American College of Emergency Physicians to recognize hospitals for higher levels of geriatric emergency care; there are now more than 170 accredited GEDs around the world.^2^

Previous studies demonstrated that geriatrics-focused emergency care quality improvement efforts administered by transitional care nurses (TCNs) who were trained in geriatric-focused assessments to deliver care addressing needs of older adults were associated with reduced risk of hospital admission^3^ and 30-day readmissions.^4^ Other programs focused on interventions or initiatives targeting older ED patients immediately after ED discharge or early during their hospital admission have also been associated with reduced risk of subsequent readmission and decreased lengths of stay.^5,6,7,8,9^ Although many hospitals and health care systems have incorporated GED initiatives targeting older ED patients as a part of their emergency care, most have not. Demonstration of the value of GED innovations with a cost analysis would yield insight into the direct economic value for patients, hospitals, health systems, and payers.

To our knowledge, no study has quantified the change in total costs per patient associated with an embedded GED care program. The purpose of this study was to evaluate total Medicare payer costs per beneficiary seen by a TCN and/or a social worker (SW) administering GED initiatives compared with costs for patients not seen by either a TCN or an SW during the same study period at 30 days and 60 days after an index ED visit.

## Methods

### Design, Setting, and Participants

In this prospective cross-sectional study, cost analyses were performed using data on unique Medicare fee-for-service (FFS) beneficiaries 65 years or older who visited 1 of 2 EDs in the Geriatric Emergency Department Innovations in Care Through Workforce, Informatics, and Structural Enhancements (GEDI WISE) program during its implementation period (January 1, 2013, to November 30, 2016, for Mount Sinai Medical Center [MSMC] in New York, New York, and April 1, 2013, to November 30, 2016, for Northwestern Memorial Hospital [NMH] in Chicago, Illinois). Analyses were conducted and data prepared from August 29, 2018, to November 20, 2020. The GEDI WISE program is described in detail elsewhere.^3,4,10,11^ This study was approved by the institutional review boards at MSMC and NMH with waivers of informed consent for the cross-sectional comparisons and with a data use agreement from Medicare. The study followed the Strengthening the Reporting of Observational Studies in Epidemiology (STROBE) reporting guideline.

### Description of the Treatment

Treatment was defined as being seen by a TCN or an SW trained specifically for the GEDI WISE program at the patient’s first ED visit (index ED visit). As described in previous studies,^3,4^ the TCN or SW targeted patients for geriatric syndromes that would be responsive to an ED-based transitional care program. The TCN was often the first to screen the patient in the ED, and they performed comprehensive geriatric, emergency care–specific assessments that were part of the GEDI WISE program. Treatment was initiated based on needs revealed during the GEDI WISE patient assessments, if requested by ED clinicians, and when the TCN or SW were available in the ED. A list of the 10 most common interventions or activities completed by the TCN or SW is available in eTable 1 in the Supplement. The comparison group (usual care) included beneficiaries never seen by either a TCN or an SW during the study period; the first ED visit was evaluated.

### Data Sources

Medicare research identifiable files (RIF) contain person-specific data on Medicare providers, beneficiaries, and recipients, including individual identifiers that allowed us to link the data to administrative patient information.^12^ We used Social Security number, first name, last name, date of birth, and Medicare beneficiary's health insurance claim numbers to crosslink the data at the patient level. The Master Beneficiary Summary File contains beneficiary enrollment information, including health maintenance organization, Medicaid eligibility, date of enrollment, type of enrollment (Part A for hospital inpatient or Part B for physician visits, preventive services, laboratory tests, medical equipment and supplies, and additional resources). To account for total costs of care per beneficiary, we included the following Medicare FFS claims: inpatient (claims submitted by inpatient hospital health care professionals for reimbursement of facility costs), outpatient (claims submitted by institutional outpatient health care professionals), carrier (claims submitted by health care professionals, including physicians, physician assistants, clinical social workers, and nurse practitioners), durable medical equipment (DME) (claims submitted by DME suppliers to the DME Medicare administrative contractor), home health agency (claims submitted by Medicare home health agency providers for reimbursement of home health–covered services), hospice (claims submitted by Medicare hospice providers), and skilled nursing facility (claims submitted by skilled nursing facility institutional providers covered by Medicare). Information about each of these files and their data documentation are further described on the Centers for Medicare & Medicaid Services Research Data Assistance Center data support website.^13^

To create our Medicare total costs data set, we created binary indicators of Medicare patient enrollment for at least 12 months before the index visit and indicators of Medicaid eligibility using information from the Master Beneficiary Summary File. We then linked claims cost data from the outpatient, inpatient, carrier, DME, home health agency, hospice base, and skilled nursing facility files to create a master data set for each hospital in the study. We used the Charlson Comorbidity coding algorithm for *International Classification of Diseases, Ninth Revision, Clinical Modification* and *International Statistical Classification of Diseases, Tenth Revision, Clinical Modification* codes, as described by Quan et al,^14^ to compute the 17 comorbidities used to classify patient diagnosis history and to calculate a composite score.

### Entropy Balance

We used entropy balancing to account for differences in patient characteristics associated with both costs and TCN or SW (treatment) interaction, which could bias the estimated association of treatment with costs. Entropy balancing is a data-preprocessing method that facilitates creation of treatment and comparison groups that have similar covariate distributions. This method makes observed characteristics of treatment and comparison groups for each site as similar as possible based on observed characteristics other than receipt of treatment. Entropy balancing assigns a scalar weight to each comparison unit such that the reweighted comparison groups satisfy a set of imposed balance constraints and maximize the balance between moments of the covariate distributions in each group.^15^ As a result, the standardized mean difference in covariates between the treatment and comparison groups becomes less than 1%.

### Outcome Measures

The primary outcome evaluated was prorated total Medicare expenditures per beneficiary over 30 and 60 days from the index day of their ED encounter. Medicare expenditures were calculated summing all payments from Medicare claims in dollars inclusive of hospital admissions or discharges from the ED of all services rendered to the beneficiary as recorded in their outpatient, inpatient, carrier, DME, home health services, hospice, and skilled nursing facility claim files.

### Risk Factors

Covariates were selected based on characteristics likely to be associated with the outcome of cost and those used in previous analyses evaluating transitional care nurse impact.^3^ Covariates included in the entropy balance algorithm were age (as a continuous variable using mean and variance); dichotomous indicators of sex; race/ethnicity groups (White, Black, Asian, Hispanic, and other race); indicator for arrival to the ED on a weekday; indicator for arrival to the ED during the evening; patient care occurring in a dedicated GED area; prior hospital discharge within 30 days; chief concerns in the following categories: pain, fall (of all types), difficulty breathing, weakness, altered mental status, and psychiatric or behavioral issues; Emergency Severity Index (ESI)^16^ (2, 3, and 4 or 5); the Identification of Seniors at Risk Score groups^17^ (0, 1, 2, 3, 4, 5 or 6, and missing); and Charlson Comorbidity Score^14^ (0, 1, 2, 3, and ≥4). These risk factors were included in both the entropy balance algorithm and the cost analysis regression models to account for potential covariate imbalance that could remain after entropy balancing, allowing for doubly robust estimation.^18,19^ The cost analysis regression model also included a binary indicator for concurrent Medicaid status. Because MSMC is a part of a Medicare Shared Savings Program accountable care organization (NMH is not), we also included an accountable care organization covariate for MSMC beneficiaries in the outcome model.

### Exclusions

We excluded from the analyses patients who left the ED against medical advice, left without being seen, left before treatment was completed, died, had age recorded as older than 118 years (this age was assumed to be erroneously reported), had an ESI of 1 (these patients were acutely ill and not routinely evaluated by the TCN and/or the SW in the ED), had missing ESI documentation, or had been covered by Medicare FFS for less than 12 months before the study start. Of the reasons for exclusion from the sample, less than 1% accounted for the initial cohort. The only reason accounting for greater than 1% of exclusions across treatment and control groups was FFS coverage for less than 12 months. The direction of this variance was different for MSMC and NMH. Nonetheless, we only included individuals with full coverage to allow for full capture of beneficiary health care use and Medicare total claims.

### Statistical Analysis

We set a priori levels of significance at *P* < .10 for bivariate and univariate analyses, and at *P* < .05 for multivariate regression analyses. Hypothesis tests were 2-sided. Model covariates were selected based on those found to have significance in bivariate analyses or of construct validity that were not highly collinear. Analyses were conducted using Stata SE, version 16.1 (StataCorp LLC).

We fitted generalized linear regression models with various family distribution and link combinations commonly used with skewed outcomes: gamma-log, gamma-power, Poisson-log, and Poisson-power. The gamma-log model was selected because it has the lowest Akaike information criterion.^20^ For each site, we estimated the mean incremental association of TCN or SW care with prorated Medicare expenditures among the treatment group. By site, multiple generalized linear regressions were completed to incrementally estimate the average treatment effect on the treated, which was the effect on Medicare expenditures for patients receiving care from a TCN and/or an SW compared with Medicare expenditures for the weighted comparison group.

Because both TCN and SW care were available concurrently and ED patients were eligible to be seen by either, our primary analysis modeled the treatment cohort as patients who received care from a TCN or an SW or both compared with patients receiving usual care (not from a TCN or an SW). We performed additional sensitivity analyses of cost variation comparing patients who received TCN treatment only, SW treatment only, and both TCN and SW treatment compared with usual care. In addition, to evaluate the subsequent outcome of death and the outcome of total costs of care, we performed a subset of analyses excluding those who died after their index ED encounter. Results are available in eTables 2 and 3 in the Supplement.

## Results

During the evaluation period, a total of 24 839 unique Medicare FFS beneficiaries were seen at both EDs (11 218 [45.2%] at MSMC and 13 621 [54.8%] at NMH). Of 4041 patients seen in a GED, 1947 patients at MSMC (17.4%) and 2094 (15.4%) at NMH were initially treated by either a GED TCN and/or a GED SW. The mean (SD) age of beneficiaries at MSMC was 78.8 (8.5) years and at NMH was 76.4 (7.7) years. Most patients at both hospitals were female (6821 [60.8%] at MSMC and 8023 [58.9%] at NMH) and White (7729 [68.9%] at MSMC and 9984 [73.3%] at NMH). There were differences in ESI level across sites, with most patients at MSMC (6669 [59.4%]) categorized as having an ESI level of 3 and most NMH patients (7204 [52.9%]) categorized as having an ESI level of 2. The Table provides a summary of demographic and clinical characteristics comparing the treatment group, the comparison group, and the weighted comparison group after entropy balance weighting for each hospital. The most common chief concern treated at both sites was fall-related injury (280 [14.4%] at MSMC and 272 [13.0%] at NMH). A total of 389 patients at MSMC (20.0%) were members of its accountable care organization. After entropy balancing, the standardized differences between the 2 groups were reduced to almost 0 for all covariates at both MSMC and NMH (Figure).

**Table.** Characteristics of the Treatment and Comparison Groups Before and After Entropy-Balanced Weighting^a^

Variable	Mount Sinai Medical Center (n = 11 218)^b^			Northwestern Memorial Hospital (n = 13 621)^c^		
	Treatment group (n = 1947)	Comparison group (n = 9271)	Weighted comparison group (n = 9271)	Treatment group (n = 2094)	Comparison group (n = 11 527)	Weighted comparison group (n = 11 527)
Age, mean (SD)	81.4 (8.7)	78 (8.4)	81.4 (8.8)	80.5 (8.1)	75.7 (7.4)	80.5 (8.5)
Male	605 (31.1)	3793(40.9)	3793(40.9)	770 (36.8)	4820 (41.8)	4820 (41.8)
Race/ethnicity						
White	1104 (56.7)	6626 (71.5)	6626 (71.5)	1400 (66.9)	8588 (74.5)	8588 (74.5)
Black	583 (29.9)	1595 (17.2)	1595 (17.2)	603 (28.8)	2214 (19.2)	2214 (19.2)
Hispanic	171 (8.8)	484 (5.2)	484 (5.2)	30 (1.4)	162 (1.4)	162 (1.4)
Asian	28 (1.4)	224 (2.4)	224 (2.4)	32 (1.5)	185 (1.6)	185 (1.6)
Other^d^	61 (3.1)	342 (3.7)	342 (3.7)	29 (1.4)	378 (3.3)	378 (3.3)
Emergency Severity Index						
2	420 (21.6)	3385 (36.5)	3385 (36.5)	1118 (53.4)	6092 (52.8)	6092 (52.8)
3	1384 (71.1)	5287 (57.0)	5287 (57.0)	680 (38.1)	3990 (34.6)	3990 (34.6)
4 or 5	143 (7.3)	599 (6.5)	599 (6.5)	178 (8.5)	1445 (12.5)	1445 (12.5)
Chief concern						
Altered mental status	57 (2.9)	287 (3.1)	287 (3.1)	63 (3.0)	197 (1.7)	197 (1.7)
Difficulty breathing	108 (5.5)	685 (7.4)	685 (7.4)	125 (6.0)	693 (6.0)	693 (6.0)
Falls	281 (14.4)	552 (6.0)	552 (6.0)	272 (13.0)	878 (7.6)	878 (7.6)
Pain	247 (12.7)	820 (8.8)	820 (8.8)	271 (12.9)	1327 (11.5)	1327 (11.5)
Psychiatric	32 (1.6)	141 (1.5)	141 (1.5)	55 (2.6)	264 (2.3)	264 (2.3)
Weakness	113 (5.8)	433 (4.7)	433 (4.7)	92 (4.4)	277 (2.4)	277 (2.4)
Charlson Comorbidity Index score						
0	715 (36.7)	3423 (36.9)	3423 (36.9)	596 (28.5)	5798 (50.3)	5798 (50.3)
1	484 (24.9)	1826 (19.7)	1826 (19.7)	371 (17.7)	2089 (18.1)	2089 (18.1)
2	324 (16.6)	1420 (15.3)	1420 (15.3)	286 (13.7)	1301 (11.3)	1301 (11.3)
3	201 (10.3)	924 (10.0)	924 (10.0)	213 (10.2)	817 (7.1)	817 (7.1)
≥4	223 (11.5)	1678 (18.1)	1678 (18.1)	628 (30.0)	1522 (13.2)	1522 (13.2)
Discharged from hospital in prior 30 d	202 (10.4)	44 (0.5)	44 (0.5)	324 (15.5)	428 (3.7)	428 (3.7)
ED visit, night or weekend presentation	631 (32.4)	3608 (38.9)	3608 (38.9)	407 (19.4)	4836 (42.0)	4836 (42.0)
ISAR						
0	101 (5.2)	860 (9.3)	860 (9.3)	127 (6.1)	2819 (24.5)	2819 (24.5)
1	444 (22.8)	3021 (32.6)	3021 (32.6)	305 (14.6)	4143 (35.9)	4143 (35.9)
2	352 (18.1)	1559 (16.8)	1559 (16.8)	298 (14.2)	1842 (16.0)	1842 (16.0)
3	311 (16.0)	1123 (12.1)	1123 (12.1)	680 (32.5)	991 (8.6)	991 (8.6)
4	256 (13.2)	800 (8.6)	800 (8.6)	438 (20.9)	675 (5.9)	675 (5.9)
5 or 6	157 (8.1)	384 (4.1)	384 (4.1)	216 (10.3)	308 (2.7)	308 (2.7)
Missing	326 (16.7)	1524 (16.4)	1524 (16.4)	30 (1.4)	749 (6.5)	749 (6.5)
ED visit in geriatric ED structural environment	1032 (53.0)	3464 (37.4)	3464 (37.4)	909 (43.4)	4447 (38.6)	4447 (38.6)
Medicaid	704 (36.2)	2184 (23.6)	2184 (23.6)	334 (16.0)	1122 (9.7)	1122 (9.7)
ACO participant	389 (20.0)	1434 (15.5)	1434 (15.5)	NA	NA	NA

Abbreviations: ACO, accountable care organization; ED, emergency department; ISAR, Identification of Seniors at Risk Score; NA, not applicable.

^a^ Data are presented as number (percentage) of patients unless otherwise indicated. Treatment included consultation with a transitional care nurse or social worker trained for the Geriatric Emergency Department Innovations in Care Through Workforce, Informatics, and Structural Enhancement program at a beneficiary’s first ED visit. The comparison group included beneficiaries who were never seen by either a transitional care nurse or a social worker during the study period. The weighted comparison groups are the comparison group after entropy-balanced weighting.

^b^ Data are from January 1, 2013, to November 30, 2016.

^c^ Data are from April 1, 2013, to November 30, 2016.

^d^ Other included American Indian, mixed race/ethnicity, and unknown race/ethnicity.

**Figure.**
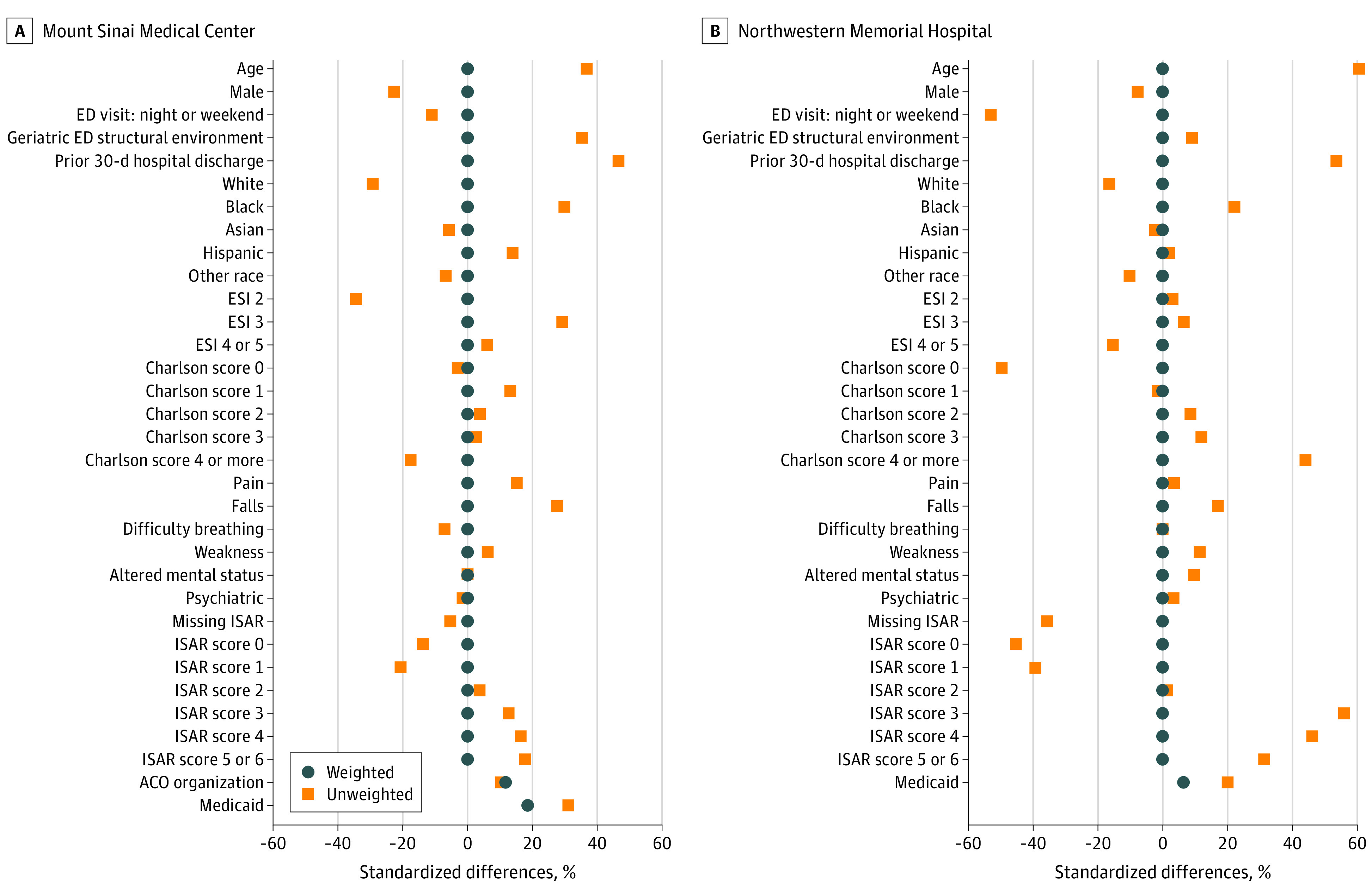
Entropy Covariate Balancing Across Treatment and Comparison Groups at Mount Sinai Medical Center and Northwestern Memorial Hospital ACO indicates accountable care organization; ED, emergency department; ESI, Emergency Severity Index; and ISAR, Identification of Seniors at Risk Score.

Regression models demonstrated an association of lower total Medicare expenditures with treatment compared with the weighted comparison group at 30 and 60 days after the index ED visit. Treatment had a significant association with savings in total Medicare expenditures per beneficiary of $2436 (95% CI, $1760-$3111; *P* < .001) in the MSMC cohort and $2905 (95% CI, $2378-$3431; *P* < .001) in the NMH cohort in the 30 days after the index ED visit. These associations of treatment with savings in total Medicare expenditures continued to be statistically significant up to 60 days after the index ED visit (mean savings per beneficiary: MSMC, $1200 [95% CI, $231-$2169; *P* = .02]; NMH, $3202 [95% CI, $2452-$3951]; *P <* .001).

Our results were robust to sensitivity analyses using different specifications of cost models. At MSMC, results were robust to different definitions of treatment groups. For beneficiaries at MSMC, there were savings at 30 days with all definitions of treatment groups: TCN only, $3606 (95% CI, $2838-$4474); SW only, $3428 (95% CI $2724-$4151); and both TCN and SW, $4768 (95% CI, $4008-$5528). These savings persisted through 60 days. For beneficiaries at NMH, we observed 30-day cost savings in the TCN-only group ($4527; 95% CI, $3981-$5073) and the TCN and SW group ($2105; 95% CI, $1546-$2665). However, when treatment was restricted to SW-only encounters, treatment was associated with increased costs ($1808; 95% CI, $1153-$2463). These cost patterns for NMH persisted through 60 days. When patients who died after the ED visit were excluded, we observed an association between treatment and cost savings at both hospitals at 30 days (MSMC, $2284 [95% CI, $1321-$3347]; NMH, $1229 [95% CI, $601-$1857]) (eTables 2 and 3 in the Supplement).

## Discussion

Among Medicare FFS beneficiaries, receipt of care from GED TCNs and/or GED SWs was associated with lower total costs of Medicare expenditures compared with not receiving care from these specialists. Medicare savings per beneficiary ranged from $2436 (at MSMC) to $2905 (at NMH) in the immediate 30 days after an index ED visit. These total cost savings continued to be sustained at 60 days, ranging from $1200 (at MSMC) to $3202 (at NMH). Even when stratifying analyses and excluding beneficiaries who died after the index encounter, treatment was associated with cost savings, although savings were reduced at 30 days at both sites and at 60 days at only 1 site.

To our knowledge, this study is the first to evaluate the association of a GED program with total costs of care per patient from a payer perspective. A strength of our evaluation was our ability to calculate total Medicare costs per beneficiary by linking hospital and clinical data for ED patients to their total Medicare claims. The hospital and clinical data allowed for the identification of ED patients seen by GED personnel and for weighted comparison with a group not receiving GED care. Quantified Medicare costs by beneficiary are the sum of all individual service payments (eg, outpatient, carrier, hospice, and DME) and those packaged under predetermined rates or partial hospitalization payments (eg, inpatient, skilled nursing facilities). By using all Medicare claims found for each beneficiary, we were able to calculate the estimated total cost savings per beneficiary among patients who were treated by a GED-trained TCN and/or SW. The difference between those seen by a TCN or an SW and those not seen provided a projected cost analysis of the association of embedded GED programs with Medicare expenditures and could be used when considering the bundled value and potential payer reimbursement per patient for such programs. For our evaluations, the association of cost savings with Medicare expenditures was consistently observed when the beneficiary was seen by a TCN.

Although geriatric emergency care programs are not currently reimbursed by any health care payers, previous studies^3,4,10^ have shown positive associations of GED programs with clinical outcomes, including decreased hospitalizations, intensive care unit admissions, 30-day hospitalizations, and costs to Medicare. In the present study, treatment was significantly associated with reduced costs per patient. The value and incentivization of such models of care may be considered for payment to facilitate integration and optimization of current emergency care practices for older adults. Hospitals and clinicians who incorporate these integrated programs in their EDs to care for geriatric patients should receive the benefit of shared savings by meeting these Triple Aim objectives.^21^

The cost savings found in this study were likely associated with a change in health care utilization and trajectory for the treated beneficiaries. In addition to GEDI WISE studies,^3,4,10^ which have shown decreases in health services use, other studies^5,6,7,8,9,22,23,24^ have also demonstrated a reduced likelihood of admission associated with GED programs. Hospitalizations are costly. We believe that the ability to provide enhanced transitions of care planning in the ED, when coupled with care by a TCN, is cost saving. We hypothesized that the association of lower Medicare expenditures with care from a TCN and/or an SW may be attributable to the reduction in avoidable patient admissions. This reduction in turn may have been associated with a reduced risk of hospital-related iatrogenic complications and functional decline, delaying nursing home admissions or decreasing the risk of institutionalization.

Of note, in sensitivity analyses for 1 of the hospitals, care by an SW alone was associated with increased Medicare costs during at 30 and 60 days after the index visit. The group of patients seen by both a TCN and an SW may have been substantially different from those who were seen by only a TCN or an SW. At the time of GEDI WISE implementation, the role of the SW at that hospital was relatively novel and the opportunity to integrate and coordinate care transitions for ED patients may have been associated with improved access to health and community care access. Because this was not a randomized clinical trial comparing patients seen by neither, one, or both types of health care workers, the cost savings may not be directly comparable. Nonetheless, the ability to provide enhanced transitions of care planning in the ED when coupled with care by a TCN was associated with cost savings. These changes in care trajectory may be reflected in the total Medicare cost savings at 30 and 60 days after the index ED visit.

### Limitations

This study has limitations. First, this was an observational study comparing patients seen by a TCN and/or an SW and those not evaluated by these GED-focused personnel. Although entropy balance and multivariable regression modeling were used to account for observable selection bias, there may still have been unobserved confounders associated with being treated and our cost outcomes. Although there may be concern of greater imbalance for variables less represented in the unweighted control group, the sample size was sufficient to generate weights for balancing with all variables included (eTable 4 in the Supplement). A randomized clinical trial would provide better balance with unobserved confounders; the observational design of the present study may provide useful information about associations between treatment and cost and savings and leveraged data from a pragmatic, real-world clinical implementation program. Second, the data set and cohort were limited to Medicare FFS beneficiaries with greater than 12 months of coverage. We did not include patients with partial FFS coverage (<12 months), those with Medicare Advantage, or those with other health care payer plans. Third, the degree to which the TCN or SW provided and facilitated geriatric-focused care per patient was not measured. These differences, along with variation in implementation of geriatric emergency care programs at the 2 sites, patient population, hospital and community resources, and 1 of the sites being part of an accountable care organization may account for the variation in cost savings at 30 and 60 days between the hospitals. Thus, our analyses were stratified by site and not pooled. In addition, our study did not include or evaluate implementation costs at hospitals using the GEDI WISE program. Our estimates reflect costs to Medicare for medical care reimbursement per patient. These results may not be generalized to other sites without geriatrics-focused TCNs or SWs in the ED setting or to non-Medicare beneficiaries. These findings provided an estimated dollar value of associated savings per patient among treatment recipients and the projected total costs of care for Medicare.

## Conclusions

Innovative models of care have been implemented over the past decade to improve the quality of emergency care, improve health outcomes, and reduce costs for older ED patients. In this study, GED care from a TCN and/or an SW was associated with lower total Medicare costs at 30 and 60 days after the index ED visit. The estimated cost savings projected in this study may be used by health care payers when considering savings for potential reimbursement models that are associated with GED programs.

## Notes

Supplement. **eTable 1.** Top 10 Interventions by the GEDI WISE Transitional Care Nurse or Social Worker **eTable 2.** Sensitivity Analyses With Different TCN or SW Treatment **eTable 3.** Sensitivity Analyses Excluding Beneficiaries Who Subsequently Died After the Index ED Encounter **eTable 4.** Entropy Balancing Weight Distribution
