# Errors in the Table

**DOI:** 10.1001/jamanetworkopen.2021.7149

**Published:** 2021-03-29

In the Original Investigation titled “Association of a Geriatric Emergency Department Innovation Program With Cost Outcomes Among Medicare Beneficiaries,”^1^ published March 1, 2021, the *n* values and all data in the weighted comparison group columns were replaced in the Table. Also, in the Northwestern Memorial Hospital treatment group column, the number of patients with an Emergency Severity Index of 3 was changed from 680 to 798. This article has been corrected.
