# Nonauthor Collaborator Names Added to a Supplement

**DOI:** 10.1001/jamanetworkopen.2021.17178

**Published:** 2021-06-15

The Original Investigation titled “Association of a Geriatric Emergency Department Innovation Program With Cost Outcomes Among Medicare Beneficiaries,”^1^ published March 1, 2021, has been corrected to include the nonauthor collaborator names in a supplement.

## References

[1] Hwang U, Dresden SM, Vargas-Torres C, ; Geriatric Emergency Department Innovations in Care Through Workforce, Informatics, and Structural Enhancement (GEDI WISE) Investigators. Association of a geriatric emergency department innovation program with cost outcomes among Medicare beneficiaries. JAMA Netw Open. 2021;4(3):e2037334. doi:10.1001/jamanetworkopen.2020.37334 33646311 PMC7921898

