# Supplementary material for: Association of a Geriatric Emergency Department Innovation Program With Cost Outcomes Among Medicare Beneficiaries
Source: JAMA Netw Open. 2021 Mar 1;4(3):e2037334. doi: 10.1001/jamanetworkopen.2020.37334 (PMC7921898; doi:10.1001/jamanetworkopen.2020.37334)
Supplement: Supplement. — eTable 1. Top 10 Interventions by the GEDI WISE Transitional Care Nurse or Social Worker eTable 2. Sensitivity Analyses With Different TCN or SW Treatment eTable 3. Sensitivity Analyses Excluding Beneficiaries Who Subsequently Died After the Index ED Encounter eTable 4. Entropy Balancing Weight Distribution [file jamanetwopen-e2037334-s001.pdf]

Supplemental Online Content

Hwang U, Dresden SM, Vargas-Torres C, et al; Geriatric Emergency Department Innovations in Care Through Workforce, Informatics, and Structural Enhancement (GEDI WISE) Investigators. Association of a geriatric emergency department innovation program with cost outcomes among Medicare beneficiaries. *JAMA Netw Open.* 2021;4(3):e2037334. doi:10.1001/jamanetworkopen.2020.37334

eTable 1. Top 10 Interventions by the GEDI WISE Transitional Care Nurse or Social Worker

eTable 2. Sensitivity Analyses With Different TCN or SW Treatment

eTable 3. Sensitivity Analyses Excluding Beneficiaries That Subsequently Died After the Index ED Encounter

eTable 4. Entropy Balancing Weight Distribution

This supplemental material has been provided by the authors to give readers additional information about their work.

© 2021 Hwang U et al. *JAMA Network Open.*

Supplement Table 1. Top 10 Interventions by the GEDI WISE Transitional Care Nurse or Social Worker[3,4,10,11] :

1. Risk assessment for adverse outcomes from the ED.
2. Risk assessments for cognitive impairment and delirium.
3. Risk assessments and interventions to decrease falls and improve mobility, consult or refer to physical therapy when appropriate.
4. Functional assessments, consult or refer to occupational therapy when appropriate.
5. Evaluation of polypharmacy and potentially inappropriate medication use, consult ED pharmacist when appropriate.
6. Coordination for direct admission from ED to skilled nursing facilities or subacute rehabilitation.
7. Transportation coordination to and from ED to home.
8. Coordination of care transitions with outpatient evaluation and initiating referrals with home care agencies to ensure home safety for discharged patients.
9. Goals of care, advanced care planning discussions with palliative care.
10. Follow-up calls for discharged patients.

© 2021 Hwang U et al. *JAMA Network Open.*

Supplement Table 2. Sensitivity analyses with different TCN or SW treatment

**Mount Sinai Medical Center, Period Jan 1, 2013 – Nov 30, 2016**						
**Treatment Group Average Incremental Effect (dollars per beneficiary)**	GLM Gamma Log Model Average Treatment on the Treated Cost at 30 Day	95% Confidence Interval	p-value	GLM Gamma Log Model Average Treatment on the Treated Cost at 60 Day	95% Confidence Interval	p-value
TCN-Only (n=9,312)	-3,606	(-4,374 - -2,838)	<0.0001	-3,224	(-4,259 - -2,198)	<0.0001
SW-Only (n=10,285)	-3,428	(-4,151 - -2,724)	<0.0001	-2,414	(-3,416 - -1,412)	<0.0001
BOTH TCN and SW (n=9,491)	-4,768	(-5,528 - -4,008)	<0.0001	-4,749	(-5,751 - -3,748)	<0.0001

© 2021 Hwang U et al. *JAMA Network Open.*

Northwestern Memorial Hospital, Period April 1, 2013 – Nov 30, 2016						
**Treatment Group Average Incremental Effect (dollars per beneficiary)**	GLM Gamma Log Model Average Treatment on the Treated Cost at 30 Day	95% Confidence Interval	p-value	GLM Gamma Log Model Average Treatment on the Treated Cost at 60 Day	95% Confidence Interval	p-value
TCN-Only (n=11,567)	-4,527	(-5,073 - -3,981)	<0.0001	-5,956	(-6,725 - -5,187)	<0.0001
SW-Only (n=10,916)	1,808	(1,153 - 2,463)	<0.0001	3,969	(3,016 - 4,922)	<0.0001
BOTH TCN and SW (11,820)	-2,105	(-2,665 - -1,546)	<0.0001	-2,871	(-3,676 - -2,065)	<0.0001

© 2021 Hwang U et al. *JAMA Network Open.*

Supplement Table 3. Sensitivity analyses excluding beneficiaries that subsequently died after the index ED encounter

**Treatment Group**<br>**TCN and/or SW**<br>**(excluding beneficiaries that subsequently died)**<br>**Average Incremental Effect**<br>**(dollars per beneficiary)**	GLM<br>Gamma Log Model<br>Average Treatment on the Treated<br>Cost at 30 Day	95% Confidence Interval	p-value	GLM<br>Gamma Log Model<br>Average Treatment on the Treated<br>Cost at 60 Day	95% Confidence Interval	p-value
**Mount Sinai Medical Center**<br>N=9,819	-2,284	(-3,347 – -1,321)	<0.0001	-887	(-2,157 – 774)	0.23
**Northwestern Memorial Hospital**<br>N=12,627	-1,229	(-1,857 – -601)	<0.0001	-1,193	(-2,074 – -290)	0.01

© 2021 Hwang U et al. *JAMA Network Open.*

Supplement Table 4. Entropy Balancing weight distribution

MSMC Weights summary distribution

if flag_np_sw == 0

entropy balancing weights

	Percentiles	Smallest		
1%	.0261485	.0110613		
5%	.0383897	.013864		
10%	.0484718	.0148612	Obs	9,271
25%	.0748756	.0153373	Sum of Wgt.	9,271
50%	.1289704		Mean	.2100097
		Largest	Std. Dev.	.4139873
75%	.2312786	9.063615		
90%	.3990679	9.173126	Variance	.1713855
95%	.5736017	10.67716	Skewness	16.53967
99%	1.193698	16.55558	Kurtosis	434.4853

© 2021 Hwang U et al. *JAMA Network Open.*

NMH Weights summary distribution

if flag_np_sw == 0

entropy balancing weights

	Percentiles	Smallest		
1%	.0072148	.0034617		
5%	.0114003	.0041771		
10%	.0158782	.0043086	Obs	11,527
25%	.0303377	.0044867	Sum of Wgt.	11,527
50%	.0649059		Mean	.1816604
		Largest	Std. Dev.	.3656353
75%	.157953	5.593703		
90%	.4591565	6.527364	Variance	.1336892
95%	.772007	6.571352	Skewness	6.04268
99%	1.841251	6.945835	Kurtosis	62.60035

© 2021 Hwang U et al. *JAMA Network Open.*
